# The saliva microbiome of *Pan* and *Homo*

**DOI:** 10.1186/1471-2180-13-204

**Published:** 2013-09-11

**Authors:** Jing Li, Ivan Nasidze, Dominique Quinque, Mingkun Li, Hans-Peter Horz, Claudine André, Rosa M Garriga, Michel Halbwax, Anne Fischer, Mark Stoneking

**Affiliations:** 1 Department of Evolutionary Genetics, Max Planck Institute for Evolutionary Anthropology, Deutscher Platz 6, Leipzig D-04103, Germany; 2 Current address: Max Planck Independent Research Group on Population Genomics, Chinese Academy of Sciences and Max Planck Society (CAS-MPG) Partner Institute for Computational Biology, Shanghai Institutes for Biological Sciences, Chinese Academy of Sciences, Shanghai 200031, China; 3 Current address: Department of Genetics, Harvard Medical School, 77 Louis Pasteur Avenue, Boston 02115, MA, USA; 4 Division of Oral Microbiology and Immunology and Department of Medical Microbiology, RWTH Aachen University Hospital, Pauwelsstrasse 30, Aachen D-52057, Germany; 5 Lola Ya Bonobo Sanctuary, Petites Chutes de la Lukaya, Kimwenza – Mont Ngafula, Kinshasa, Democratic Republic of Congo; 6 Tacugama Chimpanzee Sanctuary, P.O. Box 469, Freetown, Sierra Leone; 7 Current address: EcoHealth Alliance, 460 West 34th Street – 17th floor, New York 10001, NY, USA; 8 Current address: Molecular Biology and Biotechnology Department, International Center for Insect Physiology and Ecology, P.O. Box 30772–00100, Nairobi, Kenya

## Abstract

**Background:**

It is increasingly recognized that the bacteria that live in and on the human body (the microbiome) can play an important role in health and disease. The composition of the microbiome is potentially influenced by both internal factors (such as phylogeny and host physiology) and external factors (such as diet and local environment), and interspecific comparisons can aid in understanding the importance of these factors.

**Results:**

To gain insights into the relative importance of these factors on saliva microbiome diversity, we here analyze the saliva microbiomes of chimpanzees (*Pan troglodytes)* and bonobos *(Pan paniscus)* from two sanctuaries in Africa, and from human workers at each sanctuary. The saliva microbiomes of the two *Pan* species are more similar to one another, and the saliva microbiomes of the two human groups are more similar to one another, than are the saliva microbiomes of human workers and apes from the same sanctuary. We also looked for the existence of a core microbiome and find no evidence for a taxon-based core saliva microbiome for *Homo* or *Pan*. In addition, we studied the saliva microbiome from apes from the Leipzig Zoo, and found an extraordinary diversity in the zoo ape saliva microbiomes that is not found in the saliva microbiomes of the sanctuary animals.

**Conclusions:**

The greater similarity of the saliva microbiomes of the two *Pan* species to one another, and of the two human groups to one another, are in accordance with both the phylogenetic relationships of the hosts as well as with host physiology. Moreover, the results from the zoo animals suggest that novel environments can have a large impact on the microbiome, and that microbiome analyses based on captive animals should be viewed with caution as they may not reflect the microbiome of animals in the wild.

## Background

A major effort is underway to categorize the human microbiome and understand the factors that can influence the distribution of microbial taxa within and among individuals [1-4], as well as to investigate evolutionary aspects of the microbiomes of different species [5-8]. Fundamental questions that remain unresolved include: the extent to which the microbiome is influenced by intrinsic/internal factors (including phylogeny, vertical transmission, host physiology, etc.) vs. extrinsic/external factors (such as diet, environment, geography, etc.); whether or not there exists a core microbiome (i.e., a set of bacterial taxa characteristic of a particular niche in the body of all humans); and the extent to which sharing of microbes between individuals can occur, either directly via transfer among individuals due to contact, or indirectly via different individuals experiencing the same environmental exposure.

Interspecies comparisons can help address some of these issues [5,8,9]. Indeed, a previous study of the fecal microbiome of wild apes found a significant concordance between microbiomes and the phylogenetic relationships of the host species [9], indicating that over evolutionary timescales, intrinsic factors are more important than extrinsic factors in influencing the composition of the great ape fecal microbiome. However, the among-individual variation in the fecal microbiome was greater than expected based purely on the phylogenetic relationships of the hosts, suggesting that extrinsic factors also play a role in generating among-individual variation. A recent study also found that different chimpanzee communities could be distinguished based on their gut microbiomes [10].

Like the gut microbiome, the oral microbiome influences human health and disease and is an important target of investigation [11], and there is extensive diversity in the saliva microbiome of human populations [12-15]. Moreover, since the saliva is in closer contact with the environment than the gut, the saliva microbiome may exhibit different patterns of variation within and between different host species than the gut microbiome. To investigate the relative importance of various factors on saliva microbiome diversity, in this study we analyzed the saliva microbiomes of chimpanzees (*Pan troglodytes)* and bonobos *(Pan paniscus)* from two sanctuaries in Africa, and from human workers at each sanctuary. We reasoned that if internal factors such as phylogeny or host physiology are the primary influence on the saliva microbiome, then the saliva microbiomes of the two *Pan* species should be more similar to one another than either is to the two human groups, and the saliva microbiomes of the two human groups should be more similar to one another. Conversely, if the saliva microbiome is mostly influenced by external factors such as geography or environment, then the saliva microbiome from each *Pan* species should be more similar to that of human workers from the same sanctuary. We also investigate the existence of a core microbiome in humans vs. *Pan*. Finally, we also studied the saliva microbiome from apes from the Leipzig Zoo, and found an extraordinary diversity in the zoo ape saliva microbiomes that is not found in the saliva microbiomes of the sanctuary animals.

## Results

**Figure 1:**
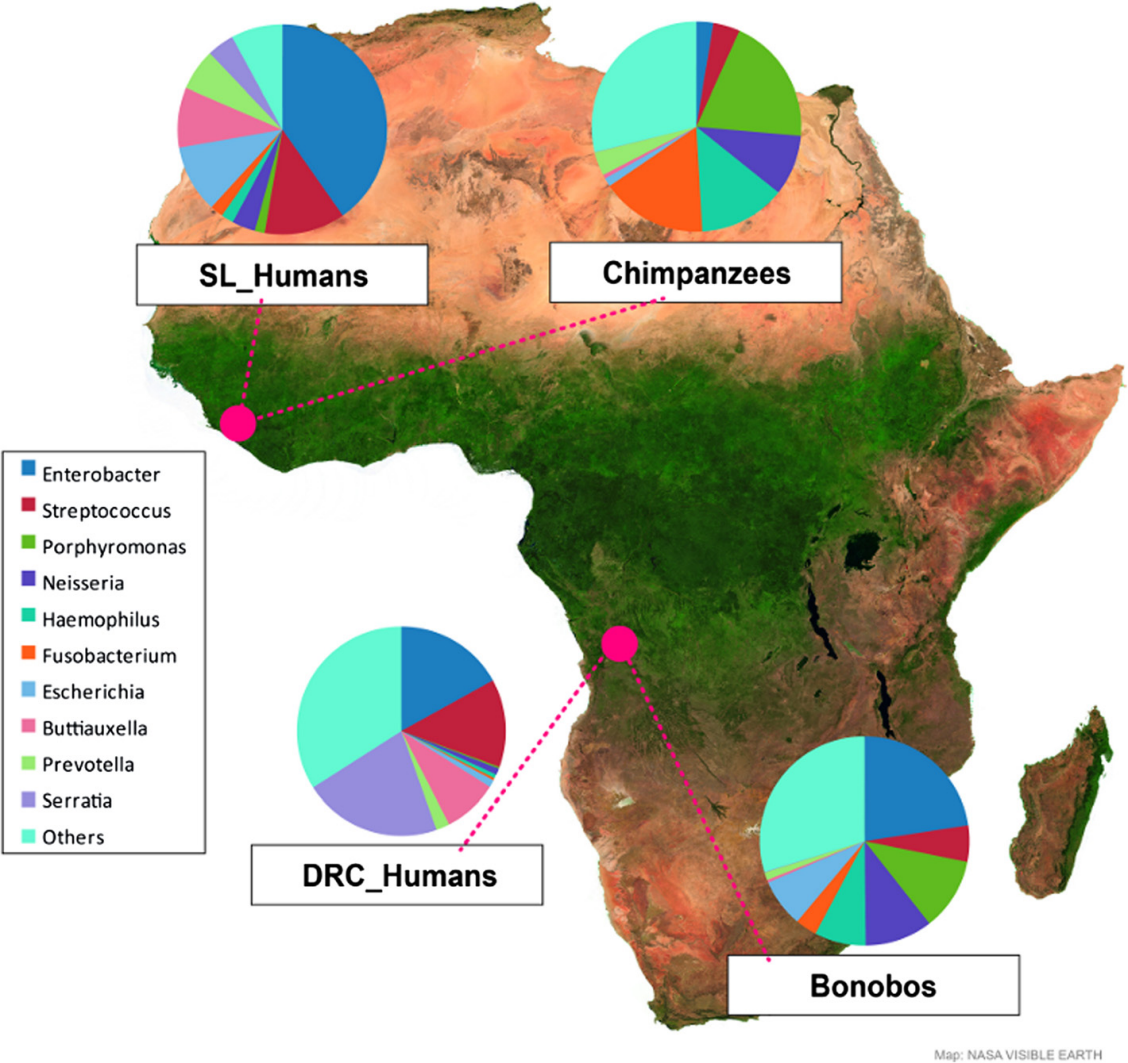
Map of the sampling locations in this study, along with pie charts of the ten most frequent bacterial genera in the saliva microbiome.

We analyzed saliva microbial diversity in 22 chimpanzees from the Tacugama Chimpanzee Sanctuary in Sierra Leone (SL), 23 bonobos from the Lola ya Bonobo Sanctuary in the Democratic Republic of the Congo (DRC), and 13 and 15 human staff members from each sanctuary, respectively (Figure 1). We amplified an informative segment of the microbial 16S rRNA gene (comprising the V1 and V2 regions) and sequenced the entire amplicon on the Genome Sequencer FLX platform. After quality filtering and removal of sequence reads less than 200 bp, there were 48,169 sequence reads in total, with the number of reads per individual ranging from 101 to 3182 (Table 1 and Additional file 1: Table S1). These were searched against the RDP database [16] in order to assign a bacterial genus to each sequence. Altogether, 93.2% of the sequences matched a previously-identified genus; 4.5% were unclassified (i.e., matched a sequence in the database for which the genus had not been classified) while 2.3% were unknown (i.e., did not match any sequence in the database above the 90% threshold value). The total number of identified genera ranged from 47 in the DRC humans to 79 in the chimpanzees (Table 1); overall, we identified 101 genera (Additional file 1: Table S1).

**Table 1:** **Statistics for the microbiome diversity in *Pan *and *Homo***

**Group**	**Number of individuals**	**Number of sequences**	**Number of OTUs**	**Unknown (%)**	**Unclassified (%)**	**Number of Genera**	**Variance between individuals (%)**	**Variance within individuals (%)**
Bonobo	23	10312	1209	3.2	4.4	69	19.1	80.9
Chimpanzee	22	14884	2394	4.1	10.0	79	11.3	88.7
Human-DRC	15	5019	731	1.0	0.5	47	36.3	63.7
Human-SL	13	17954	1797	0.8	1.1	59	28.9	71.1

Unknown (%) is the percentage of sequences that do not match a sequence in the RDP database. Unclassified (%) is the percentage of sequences that match a sequence in the RDP database for which the genus has not been classified.

To determine if the differences in number of genera observed among groups simply reflect differences in the number of sequences obtained, we carried out a rarefaction analysis, which involves subsampling different numbers of reads from each group. The results (Additional file 2: Figure S1) indicate that the two *Pan* species have similar numbers of identified genera across the different numbers of subsampled reads, and are consistently higher than the two human groups (which are similar to one another). Moreover, the number of genera detected per species/group is not related to the sample size (r = 0.60, p = 0.30). Thus, after correcting for differences in the number of reads, there are more genera detected in the saliva microbiome of the two *Pan* species than in the two human groups. However, despite the smaller number of genera detected in the two human groups, a larger fraction of the variance in their saliva microbiome is due to differences among individuals (28.9-36.3%) than is the case for the two *Pan* species (11.3-19.1%), as shown in Table 1. Overall, then, the human saliva microbiome is characterized by fewer genera, but bigger differences in composition among individuals, than is the *Pan* saliva microbiome.

**Figure 2:**
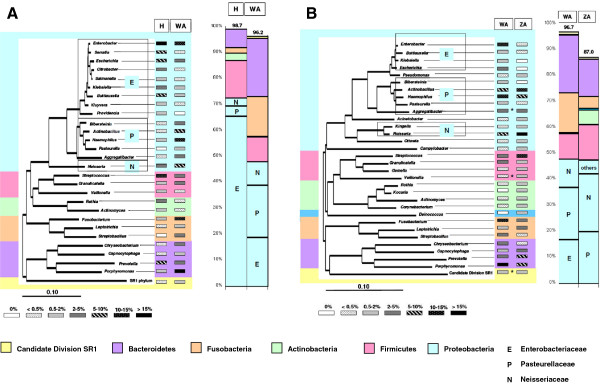
**Relative abundance of predominant genera (> 0.5%) indicated by with gray scale values with significant differences in: A, African humans (H) compared to sanctuary apes (WA); B, sanctuary apes (WA) compared to zoo apes (ZA).** Non-significant differences are indicated by asterisks. The phylogenetic tree was calculated with representative full-length sequences as implemented in the ARB program package [46] using the Jukes-Cantor correction. The scale bar represents evolutionary distance (10 substitutions per 100 nucleotides). Bacterial phyla are indicated by different colors; the vertical bars on the right of each plot indicate the relative abundance of each phylum, as marked by the colors.

A heat plot (Additional file 2: Figure S2) of the frequency of each genus in each individual indicates that the dominant genera in the saliva microbiomes of the two *Pan* species are different from those in humans. While the ten most frequent genera (accounting for 78% of all sequences) are indicated in the pie charts in Figure 1, a detailed distribution of all bacterial genera with abundances over 0.5% in at least one group is shown in Figure 2. These 28 genera accounted for 98.7% of all sequences in humans and 96.2% in the apes. The frequencies of all displayed genera were significantly different between *Pan* and *Homo* (chi-square tests, p < 0.001). The most striking differences were seen in the Gamma-*Proteobacteria* in which various genera within the family *Enterobacteriaceae* (particularly the genus *Enterobacter*) consistently dominated in humans. Conversely, a number of genera within *Pasteurellaceae* consistently dominated in the apes, along with *Neisseria* (from the Beta-*Proteobacteria*). With one exception (*Granulicatella*) genera within the phyla *Firmicutes* and *Actinobacteria* had higher abundances in humans than in apes. In contrast, genera within *Fusobacteri*a and *Bacteroidetes* exhibited higher abundances in apes compared to humans (with the exception of *Prevotella*).

Partial correlation analysis was performed in order to compare possible interactions among bacterial genera in humans with those in apes (Additional file 2: Figure S3). In agreement with the spatio-temporal model of oral bacterial colonization, which includes bacterial coaggregation in saliva and on hard tooth surfaces and soft epithelial tissues, and intergeneric metabolic interactions [17,18], significant positive correlations in humans were seen between the following pairs (Additional file 2: Figure S3A): *Fusobacterium*/*Porphyromonas*, *Fusobacterium*/*Prevotella*, *Prevotella*/*Veillonella*, *Streptococcus*/*Actinomyces,* and *Veillonella/Actinomyces.* Except for the pair *Fusobacterium/Prevotella*, no such correlations were seen within apes (Additional file 2: Figure S3B). However four significant positive correlations could be seen in both humans and apes, namely *Serratia*/*Buttiauxella*, *Fusobacterium/Leptotrichia*, *Streptococcus*/*Granulicatella*, and *Haemophilus*/*Bibersteinia*. In addition, in both humans and apes there was a tendency for genera to correlate positively with other genera from the same phylum (especially within *Proteobacteria* and Firmicutes, the two phyla with highest abundances). Within *Proteobacteria*, most genera correlated with others even from the same family (i.e. genera within *Enterobactericeae* correlate with each other and so did the genera within the *Pasteurellaceae*).

To further investigate the relationships between the *Pan* and *Homo* saliva microbiomes, we calculated Spearman’s correlation coefficient, based on the distribution of bacterial genera, between each pair of individuals. A heat plot of these correlation coefficients is shown in Additional file 2: Figure S4. The average correlation coefficient was 0.56 among bonobos, 0.59 among chimpanzees, 0.53 between bonobos and chimpanzees, and 0.55 between any two apes. The average correlation coefficient was 0.43 among DRC humans, 0.53 among SL humans, 0.46 between SL humans and DRC humans, and 0.46 between any two humans. The lower correlation coefficients among humans than among apes is in keeping with the observation above of overall bigger differences in the composition of the saliva microbiome among humans than among apes. The correlation coefficient between humans and apes was 0.34, lower than the comparisons within species; to test if the similarity in the saliva microbiome between groups from the same species was significantly greater than that between species, we carried out an Analysis of Similarity (ANOSIM). The ANOSIM analysis indicates that the within-species similarity for the saliva microbiome is indeed significantly greater than the between-species similarity (p = 0.0001 based on 10,000 permutations).

The correlation analysis also indicates that the saliva microbiomes of bonobos and chimpanzees, and of DRC humans and SL humans, are more similar to one another than any ape microbiome is to any human microbiome. Specifically, the distribution of correlations between bonobos and chimpanzees (mean = 0.53) was significantly higher (p < 0.001, Mann–Whitney U tests) than that between bonobos and staff members at the DRC sanctuary (mean = 0.30) or that between chimpanzees and staff members at the SL sanctuary (mean = 0.38). Similarly, the distribution of correlation coefficients was significantly higher (p < 0.001) between SL humans and DRC humans (mean = 0.46) than between either group of humans and apes at the same sanctuary.

**Figure 3:**
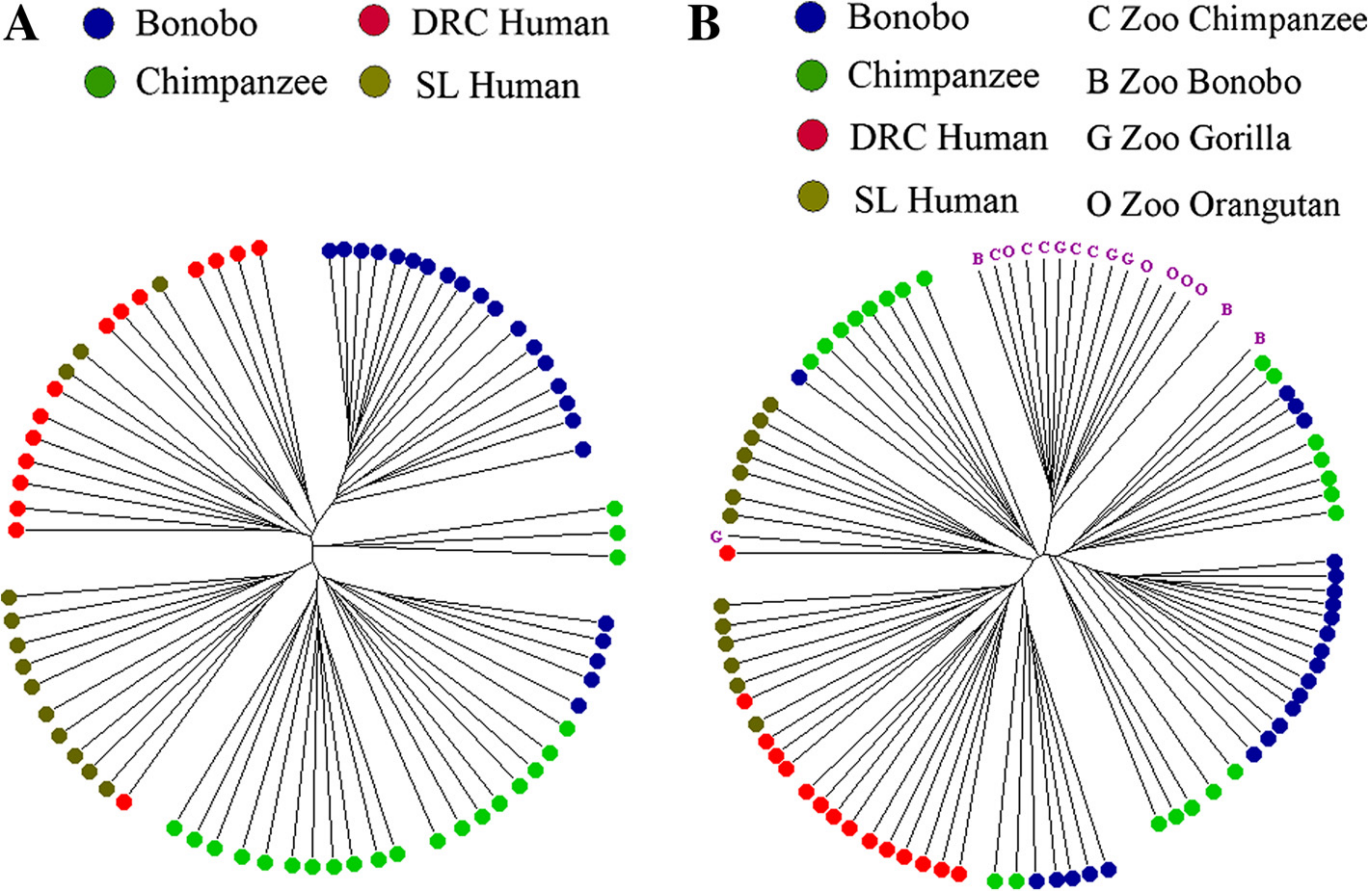
**Cluster (UPGMA) tree based on UniFrac distances. A**, Bonobos, Chimpanzees, DRC Humans, and SL Humans. **B**, including zoo apes (B = bonobo, C = chimpanzee, G = gorilla, O = orangutan).

**Figure 4:**
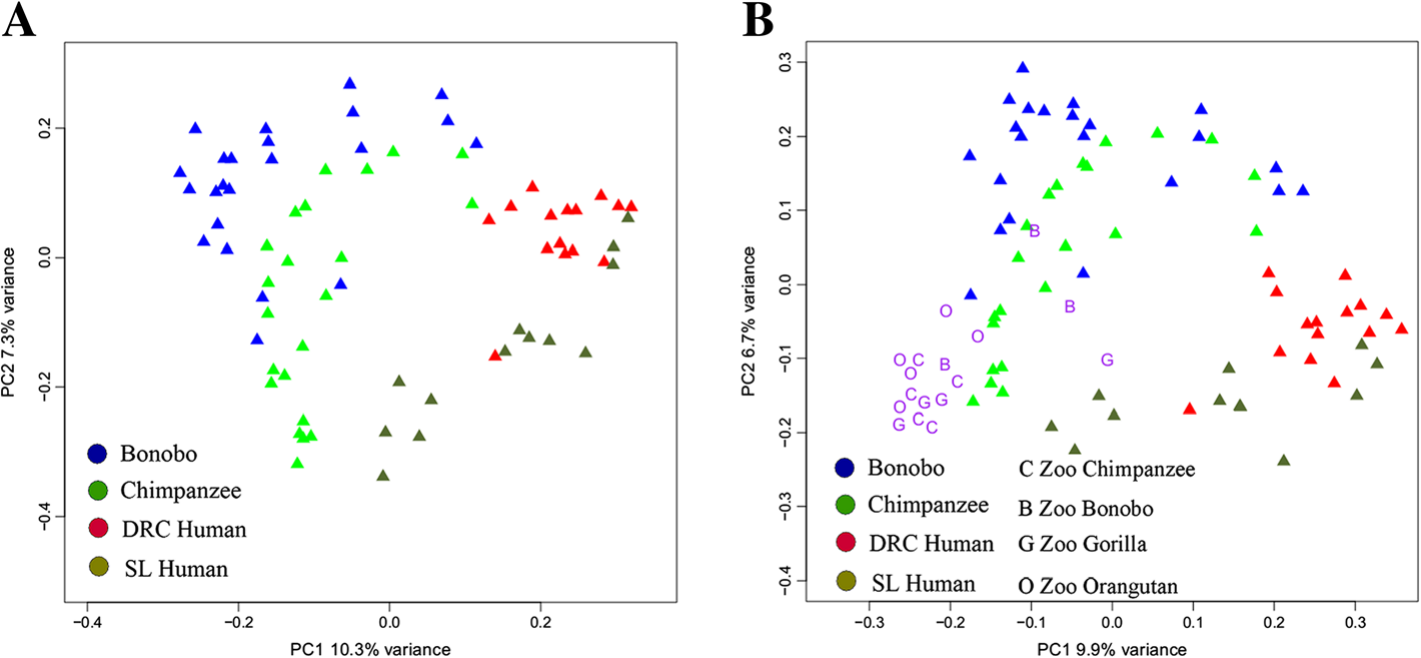
**Plots of PC1 vs. PC2, based on UniFrac distances. A**, Bonobos, Chimpanzees, DRC Humans, and SL Humans. **B**, including zoo apes (B = bonobo, C = chimpanzee, G = gorilla, O = orangutan).

We also carried out UniFrac analysis [19] to estimate the overlap in the microbiome between different individuals, and constructed a tree of the resulting UniFrac distances. In this tree (Figure 3A) the bonobos and chimpanzees appear in mostly distinct clusters, while the two human groups are more intermingled with one another. We also carried out principal component (PC) analysis of the UniFrac distances; the resulting plot of PC1 vs. PC2 (Figure 4A) is concordant with the tree in showing differences between the ape and human saliva microbiomes, although with some overlap. The UniFrac analysis thus distinguishes the saliva microbiome of the two *Pan* species from that of the two human populations, albeit not completely.

The average UniFrac distance between the two human groups is significantly larger than that between the two ape species, while the average UniFrac distance between the humans and the wild apes is significantly larger than that within either species (Additional file 2: Figure S5). As a measure of within-population diversity based on OTUs, we also calculated Faith’s Phylogenetic Diversity (PD), which is the total length of all of the branches in a phylogenetic tree that encompass the group of interest [20]. The results (Additional file 2: Figure S6) indicate that DRC humans have less diversity than bonobos (from the same sanctuary), whereas SL humans and chimpanzees have equivalent levels of PD.

**Figure 5:**
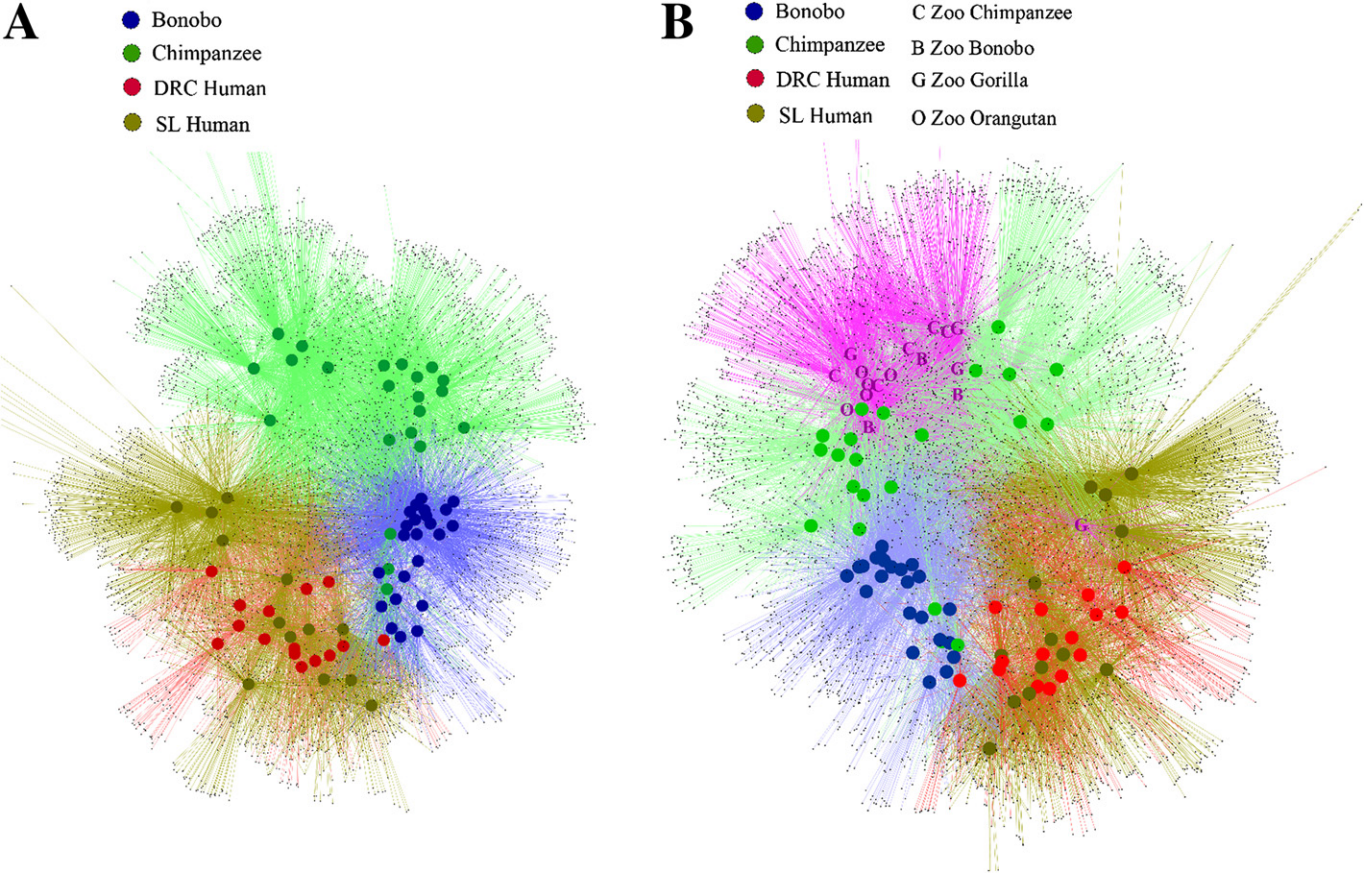
**Network analyses. A**, Bonobos, Chimpanzees, DRC Humans, and SL Humans. **B**, including zoo apes.

The UniFrac analysis summarizes the overlap in microbiomes between each pair of individuals by a single number, thereby losing information. We therefore also used a network-based approach to analyze the relationships among sequences and individuals. In this analysis, the individual sequences were first assigned to OTUs by collapsing sequences that differ by less than 3%, to avoid any influence of sequence errors. The resulting OTUs and individuals were then designated as nodes in a network, with OTUs connected to the individual(s) that they were found in. The resulting diagram (Figure 5A) completely distinguishes the microbiomes of the two *Pan* species from the two human populations. The bonobos and chimpanzees are nearly completely distinguished from one another, with three chimpanzees grouping with the bonobos (these are the same three chimpanzees that group with the bonobos in Figure 3A). Individuals from the two human groups are intermingled with one another.

We also compared the saliva microbiome from the humans and sanctuary apes to the fecal microbiome from humans and wild apes from a previous study [9]. For this analysis, we used the assignment of sequence reads to bacterial phyla, to correspond to the fecal microbiome study. The distribution of bacterial phyla in the saliva and fecal samples is provided in Additional file 3: Table S2; while overall the same phyla are abundant in both saliva and fecal samples, there are differences in the order of abundance (for example, the phylum *Firmicutes* is most abundant in fecal samples while the phylum *Proteobacteria* is most abundant in saliva samples). The average correlation coefficient for the distribution of bacterial phyla (regardless of the host species) was higher among fecal samples (average r = 0.86) and among saliva samples (average r = 0.86) than between fecal and saliva samples (average r = 0.56). Lower correlation coefficients were obtained for the comparison between fecal and saliva samples from the same species (humans: r = 0.61; bonobos: r = 0.59; chimpanzees: r = 0.59). Thus, this analysis indicates that the microbiome tends to be more similar in the same sample type (saliva or fecal) across different species than in different sample types from the same species. However, it should be noted that different individuals from different locations were analyzed for the fecal vs. saliva microbiome, and moreover different regions of the 16S rRNA molecule were analyzed. It would be desirable to further investigate this issue by analyzing the same region of the 16S rRNA molecule in fecal and saliva samples from the same individuals.

### Core microbiome

The evaluation and characterization of the core microbiome associated with a particular habitat (defined as the set of microbial OTUs that are characteristic of that habitat and thus may be important for microbiome function in that habitat) is a fundamental concern in studies of microbiome diversity [2,21,22]. This issue is complicated by the fact that there are various ways to define a core microbiome, as well as to assess whether or not a particular OTU is characteristic of an assemblage [22]. It seems reasonable to suppose that a core microbiome should be characteristic of a species (or of closely-related species); we therefore investigated the existence of a *Homo* saliva core microbiome by considering the OTUs shared by both human groups and absent in the apes, and similarly the existence of a *Pan* saliva core microbiome by considering the OTUs shared by both chimpanzees and bonobos and absent in the two human groups. We adopt a conservative approach and consider an OTU as belonging to the *Homo* core microbiome if it is present in at least one member of each human group (and absent from bonobos and chimpanzees), and as belonging to the *Pan* core microbiome if it is present in at least one chimpanzee and one bonobo (and absent from all humans). This allows for the possibility that OTUs were present in additional individuals in each species, but were not sampled (although on average 660 sequences were sampled per individual in this study, so there is a 99% chance of sampling any OTU present at a frequency of at least 0.7% in an individual).

**Figure 6:**
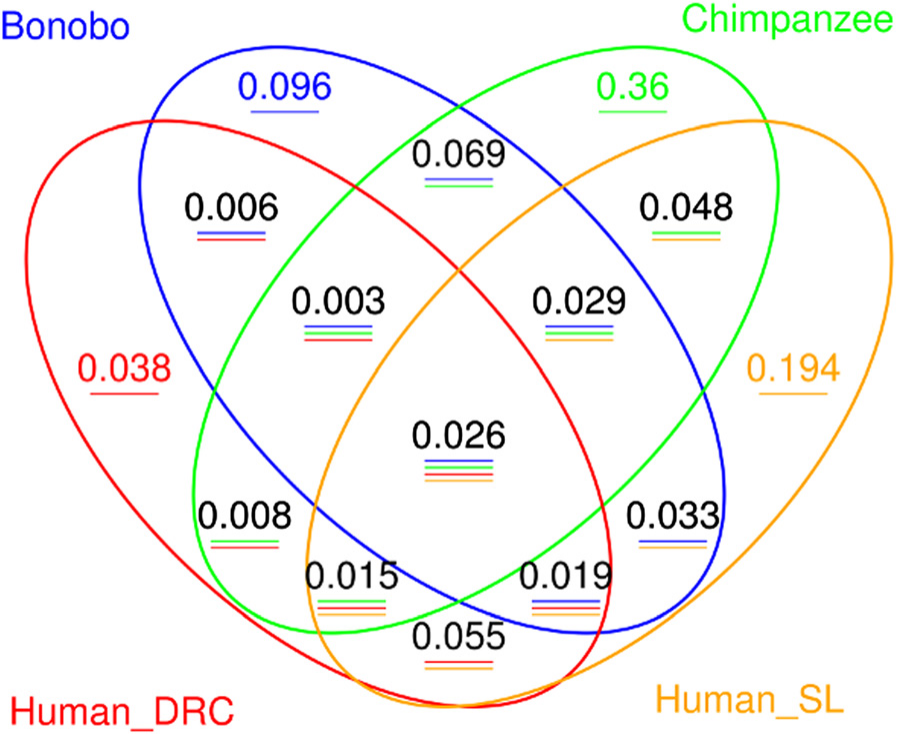
**Sharing (Venn) diagram based on OTUs in sanctuary apes and humans.** The number in each quadrant depicts the fraction of the total OTUs shared by the groups (i.e., found in at least one individual in the group) represented by that quadrant, with the colored horizontal lines further indicating the groups for each quadrant.

A Venn (sharing) diagram based on OTUs (Figure 6) shows that, based on this definition, 5.5% of the OTUs are shared by the two human groups exclusively and hence are considered the putative *Homo* core microbiome, while 6.9% of the OTUs are shared by the two *Pan* species exclusively and hence constitute the putative *Pan* core microbiome. The OTUs constituting the putative *Homo* core occurred in an average of 12.1% of the humans (range: 7.1 – 35.7%), and the average number of reads per core OTU was 7.8 (range: 2 – 116). For the putative *Pan* core, the OTUs occurred on average in 10.3% of the apes (range: 4.4 – 55.6%), and the average number of reads per core OTU was 16.0 (range: 2 – 330). Altogether, the OTUs in the putative *Homo* core microbiome comprise 11.5% of the total OTUs (and 7.9% of the total reads) for the two human groups, while the putative *Pan* core microbiome OTUs comprise 9.7% of the total OTUs (and 18.5% of the total reads) for the bonobos and chimpanzees.

We also considered the existence of a potential joint *Homo-Pan* core saliva microbiome, based on OTUs that are present in at least one individual from each of the two human groups and from each of the two ape species. As shown in Figure 6, 2.6% of the OTUs were found in at least one individual from each of the four groups. These OTUs occurred in an average of 17.6% (range 5.5 – 46.6%) of the 73 individuals in these four groups, with an average of 165.9 (range 5 – 3670) reads per OTU; this putative interspecies core saliva microbiome accounts for 38.9% of the total reads.

### Zoo apes

To determine if the above results based on sanctuary animals also hold for zoo animals, and to extend them to additional ape species, we also analyzed the saliva microbiomes from three bonobos, five chimpanzees, four lowland gorillas, and five orangutans from the Leipzig Zoo (Table 2 and Additional file 4: Table S3). The diversity in the saliva microbiome of the zoo apes was extraordinarily high, with 54 – 135 bacterial genera detected per ape species (compared to 69 – 79 genera in the sanctuary apes). Although fewer genera were detected in the saliva of zoo bonobos compared to sanctuary bonobos, rarefaction analysis (Additional file 2: Figure S1) clearly indicates that this difference is due to fewer sequencing reads for the zoo vs. the sanctuary bonobos; for similar numbers of reads, about twice as many genera are detected in the three zoo bonobos as in the 23 sanctuary bonobos. Overall, we detected 180 genera in the saliva of the 17 zoo apes (Additional file 2: Figure S2 and Additional file 4: Table S3), compared to 101 genera in the saliva of 73 apes and humans from the two sanctuaries. The elevated diversity in the zoo apes cannot be due to sample size, as the sample sizes for the zoo apes are considerably smaller than those for the sanctuary apes. Moreover, rarefaction analysis (Additional file 2: Figure S1) indicates that the elevated diversity in the zoo apes is not an artifact of differences in sequencing depth. Instead, this extraordinary diversity appears to be an inherent feature of the saliva microbiome of the zoo apes. In fact, the rarefaction analysis suggests that much diversity remains to be documented in the zoo ape saliva microbiomes, so the patterns noted below may change with additional sampling.

**Table 2:** Statistics for the microbiome diversity in zoo apes

**Species**	**Number of individuals**	**Number of sequences**	**Number of OTUs**	**Unknown (%)**	**Unclassified(%)**	**Number of Genera**	**Variance between individuals (%)**	**Variance within individuals (%)**
Bonobo	3	558	247	4.3	5.9	54	2.1	97.8
Chimpanzee	5	2263	700	8.8	4.5	135	1.7	98.3
Gorilla	4	1943	644	5.9	8.8	100	4.2	95.8
Orangutan	5	2174	562	4.9	4.3	93	0.8	99.2

Unknown (%) is the percentage of sequences that do not match a sequence in the RDP database. Unclassified is the percentage of sequences that match a sequence in the RDP database for which the genus has not been classified.

The relative abundance of the predominant genera in zoo apes vs. sanctuary apes is shown in Figure 2B. These 32 genera accounted for 96.7% of all sequences in sanctuary apes but only 87% in zoo apes. At the phylum level, sanctuary and zoo apes showed comparable relative abundances, except for the presence of the *Deinococcus* phylum in zoo apes. However differences were seen within phyla,with the most striking differences seen in the Gamma-*Proteobacteria*; zoo apes were virtually free of *Enterobacteriaceae* but instead had a much higher abundance of *Neisseria* and *Kingella*. *Pasteurellaceae* were present in roughly equal proportions in sanctuary and zoo apes. With one exception (*Granulicatella*), genera within the phyla *Firmicutes* and *Actinobacteria* had consistently higher abundances in zoo than in sanctuary apes. No consistent trend could be observed for the genera within *Fusobacteria* and *Bacteroidetes*, however overall those two phyla were more abundant in sanctuary apes (Figure 2B).

The average Spearman’s rank correlation coefficient based on the frequency of genera among pairs of individuals was 0.51 (range 0.50-0.57) within each species of zoo ape and 0.51 (range 0.49 – 0.54) between each pair of species of zoo ape. For the zoo apes, the within-species correlations are thus closer to (and in some cases even overlap) the between-species correlations, compared to the correlations for the humans vs. the sanctuary apes. Nevertheless, the ANOSIM analysis indicates that the between-species differences are significantly greater than the within-species differences for the zoo apes (p = 0.0002 based on 10,000 permutations).

To compare the saliva microbiome of the zoo apes to the humans and sanctuary apes, we calculated UniFrac distances. The tree based on UniFrac distances (Figure 3B) places 15 of the 17 zoo apes in a separate cluster (along with three of the sanctuary bonobos), while PC analysis (Figure 4B) also emphasizes the distinctiveness of the zoo ape microbiomes (irrespective of species). Nonetheless, the average UniFrac distance between zoo apes and wild apes is significantly smaller than between either ape group and humans (Additional file 2: Figure S5), indicating more similarity in the saliva microbiome among ape species than between apes and humans. Moreover, three of the four zoo ape species have higher estimates of Faith’s PD than any of the human groups or wild apes (Additional file 2: Figure S6). The network analysis of OTUs, including the zoo apes with the sanctuary apes and humans (Figure 5B), still shows largely separate clusters of the sanctuary bonobos, sanctuary chimpanzees, and the two human groups intermingled; 16 of the 17 zoo apes fall into a fourth cluster, with one zoo gorilla falling into the human group. All of these analyses indicate that the saliva microbiomes of the zoo apes are highly distinct from those of the sanctuary apes.

The data from zoo apes also provide further insights into the question of the existence of a core microbiome. Of the OTUs that comprise the putative human core saliva microbiome (found in at least one individual from each human group and absent in the sanctuary apes), 13.6% were also found in the zoo apes. Of the OTUs that comprise the putative *Pan* core saliva microbiome, 29.6% were also found in the zoo apes (20.5% in just the zoo bonobos and zoo chimpanzees). Thus, the zoo apes do share more OTUs with the putative *Pan* core microbiome than with the putative human core microbiome. In addition, 42.5% of the putative *Homo – Pan* core saliva microbiome OTUs (found in at least one individual from each human group and each *Pan* species) were also found in the zoo apes. Given the more limited sampling of zoo apes than of the sanctuary ape and human groups, these data do provide some support for the idea that these putative core OTUs are indeed widespread in humans and apes.

### OTU-sharing between species

In the above sections we demonstrated overall greater similarity between the saliva microbiome of the two *Pan* species, and between the two groups of human workers, than between the saliva microbiome of workers and apes at the same sanctuary. Here we investigate patterns of OTU-sharing in more detail, to see if there is any sharing of OTUs between apes and human workers at the same sanctuary. Such sharing could be due to either contact between the apes and humans, or independent transfer of the same OTUs from the sanctuary environment to the apes and humans at that sanctuary. Additional file 5: Table S4 lists the number and percentage of shared OTUs among the sanctuary apes, human workers, and zoo apes; a detailed listing of the OTUs in each host group/species is provided in Additional file 6: Table S5. As before, an OTU is considered to be shared if it is found in at least one member of each of the two species/groups compared. The highest amount of OTU-sharing is indeed between chimpanzees and bonobos (18.0%) and DRC and SL humans (24.2%), with less OTU-sharing between any ape and any human group (7.8 – 18.0%). The chimpanzees do share more OTUs with the SL humans at the same sanctuary (13.8%) than with the DRC humans at the bonobo sanctuary (7.8%), which could indicate a greater influence of environment/contact in this case. However, the bonobos and DRC humans share 13.7% of their OTUs, which is actually less than the fraction of OTUs (18.0%) shared between bonobos and SL humans. Overall these results do not make a compelling case for a major influence of environment/contact on the saliva microbiomes of human workers and apes at the same sanctuary.

We also investigated this issue with respect to the zoo apes, as here we have different species living in close proximity. As shown in Additional file 5: Table S4, there is on average higher OTU-sharing between the various pairs of zoo apes than between apes and humans in the sanctuaries: the average OTU-sharing between species is 20.6% for the zoo apes vs. 13.8% between apes and human workers at the same sanctuary. Thus, the zoo environment does appear to have significantly enhanced the sharing of OTUs among the different ape species.

## Discussion and conclusions

We provide here the first comparative analysis of the saliva microbiome of bonobos, chimpanzees and humans. We find greater similarity in the composition of the saliva microbiome between bonobos and chimpanzees, and between human workers at the same sanctuaries. These results suggest that internal factors, related to phylogeny or host physiology, have a more important influence on the saliva microbiome than does geography or local environment. Phylogeny (i.e., vertical transmission of the microbiome) has been previously implicated in an analysis of the fecal microbiome from wild apes [9] and is in keeping with mother-child and twin studies of the oral microbiome that found a greater role for vertical than horizontal transmission [23,24]. However, a recent study of mothers and infants found a higher correlation among the microbiomes of infants and of mothers than of infants with their mothers [25], suggesting that diet related aspects of host physiology may also play a role. Our results are compatible with either phylogeny or dietary factors related to host physiology (e.g., proportion of meat in the diet) – or both – as the primary influence(s) on the saliva microbiome. We do not find strong evidence for geography or local environment as having a major influence on the saliva microbiome; although more OTUs were shared between chimpanzees and workers at the same sanctuary than between chimpanzees and workers at the bonobo sanctuary, the opposite was true for the bonobos. Thus, even though much of the actual food sources overlap between the human workers and the apes at each sanctuary, this seems to have at best a minor effect on their saliva microbiomes. However, other potential influences on the saliva microbiome (disease status, actual individual nutrition, etc.) were not available and hence remain to be investigated.

Both the human and ape salivary microbiome was dominated by *Proteobacteria*, followed by *Firmicutes* in humans and *Bacteroidetes* in apes. *Actinobacteria* were much more dominant in apes than in humans. Those differences in phyla distribution between humans and apes are within the range that has previously been reported among humans [26]. Hence, at the phylum level the saliva microbiome of humans and apes does not differ dramatically. Within *Proteobacteria*, both humans and apes are characterized by high proportions of *Enterobacteriaceae*, which is in agreement with our previous analysis of African populations [14,15] but which stands in stark contrast to other recent oral microbiome studies that focused mainly on individuals of European ancestry [26-28]. *Enterobacteriaceae* are known to emerge in the oral cavity with increasing age and they can act as opportunist pathogens, especially in patients with debilitating diseases who are submitted to prolonged treatments with antibiotics or cytotoxic medications [29]. Although few studies have explicitly analyzed the occurrence of *Enterobacteriaceae* in the oral cavity of healthy individuals, they have been reported in nasopharyngeal swabs from northern Africans [30] and in the anterior nares of African-Americans [3]. We conclude that *Enterobactericeae* may be a consistent marker bacterial family that distinguishes African populations from other world-wide geographical regions. The reason for the higher abundance of *Enterobacteriaceae* in African populations remains unknown; knowledge of precise species would help elucidate the source of enterobacterial colonization (uptake of free-living species from plants, or introduction through consumption of fecal-contaminated food or water).

In addition to the *Proteobacteria*, most genera within the *Firmicutes*, *Actinobacteria*, *Fusobacteria* and *Bacteroidetes* were either consistently higher or lower in one group compared to the other. Such consistencies may support the concept of an ecological coherence of high bacterial taxonomic ranks, as discussed previously [31]. This means that bacterial taxa in a given phylum or family exhibit similar ecological traits, allowing the occupation of similar niches in a given host. Since obligate anaerobic bacteria (e.g., *Fusobacteria* and *Bacteroidetes*) occurred at much higher levels in sanctuary apes than in humans, differential oxygen levels might be one driving physical factor shaping the oral habitats represented by the salivary microbiome in humans and apes.

Since saliva is not considered to have its own microbiota but rather reflects the microbiome colonizing the tongue, tonsils, throat, hard and soft palate, buccal surfaces and gingivae [27], correlations between bacterial taxa might mirror interdependencies and interactions occurring at these body sites. Such interactions (which are to our knowledge unknown) might differ from recognized bacterial interactions in dental plaque or other mineralized surfaces, such as in the spatiotemporal model of oral bacterial colonization [18]. Nonetheless, the partial correlation analysis (Additional file 2: Figure S3) revealed a number of positive correlations among certain genera (including *Actinomyces*, *Fusobacterium*, *Porphyromonas*, *Prevotella*, *Streptococcus*, and *Veillonella*) that agrees with recognized dental plaque interactions, and also with a recent study that demonstrated how key oral species interact in order to grow in concert on saliva [17]. Hence, there appear to exist tight linkages among distinct bacterial taxa across various ecological oral niches. Interestingly, the lack of analogous positive correlations in apes suggests that other bacterial interactions may prevail in their oral cavity, which strengthens the overall distinctiveness of the *Pan* and *Homo* microbiomes. Conversely, there were also a number of positive correlations present in both humans and apes. Although the underlying reasons for those correlations remain unknown for now, they might indicate basic bacterial interactions that are robust across a variety of primate hosts.

Our results provide only limited support for the concept of a taxon-based core microbiome, i.e. a set of microbial OTUs which are characteristic of the saliva microbiome across a set of individuals/species, and hence may be important for the functional requirements of the saliva microbiome. A previous study that found support for a core oral microbiome (~75% of the OTUs in the study) in healthy individuals [28] was based on just three individuals; the putative core microbiome that we identified for humans as well as for apes accounts for a much smaller fraction of the OTUs in our study (12.1% and 10.3% respectively), even though we only required core OTUs to be found in at least one individual from each group/species. Although it is possible that these putative core OTUs do exist in the other individuals but at too low a frequency to be detected, the depth of sequencing in this study was sufficient to detect (with 99% probability) on average any OTU present at a frequency of 0.9% or more. Thus, even if a core saliva microbiome does exist that was not detectable in the present study, it would seem to account for at most a small fraction of the OTUs that comprise the saliva microbiome. Alternatively, it may be that the core microbiome is defined functionally rather than taxonomically, such that different OTUs are able to provide the same functionality, as has been suggested for the gut microbiome [22,32]. Indeed, the relative consistency at the phylum level across humans and apes, with variation in the specific genera within each phylum, may be consistent with a function-based core microbiome, as different genera within each phylum may be carrying out similar functions. However, further work is needed to investigate the possibility of a functional core saliva microbiome.

To extend these results to more groups and additional ape species, we also analyzed the saliva microbiomes of apes from the Leipzig Zoo. The zoo apes exhibit extraordinary diversity in their saliva microbiome that is not evident in the sanctuary apes, with over 180 bacterial genera identified in just 17 zoo apes, compared to 101 bacterial genera identified in 73 apes and human workers at the sanctuaries. Moreover, there is no consistent distinction among the saliva microbiomes of zoo bonobos, chimpanzees, gorillas, or orangutans. The results are in stark contrast to the results obtained from the sanctuary apes. Furthermore, we detect a significantly higher amount of shared OTUs among zoo apes than among the apes and human workers from the same sanctuary. It therefore appears as if the zoo environment is indeed having a significant impact on the saliva microbiome of zoo apes, which seems to contradict the conclusions based on the comparison of sancturary apes and human workers. The artificial nature of the zoo environment (in particular, the closer proximity of the zoo apes to both other apes and other species) may be responsible for this difference, but further investigation and comparisons of zoo animals with their wild counterparts are needed.

One of the most striking differences between the wild and zoo ape microbiomes was the entire absence of *Enterobacteriaceae* in zoo apes, with a correspondingly higher representation of *Neisseria* and *Kingella* instead. Apparently the zoo environment prevents *Enterobacteraceae* from steadily colonizing the oral cavity. This in turn suggests that *Enterobacteriaceae* - when not constantly introduced from the environment - are replaced by the related but truly endogenous (or highly host-associated) genera from the *Pasteurellaceae* and *Neisseriaceae* families. Hence, environment may play an important role in terms of the opportunities for particular bacteria to colonize the oral cavity.

Another striking difference between the zoo and wild ape microbiomes is the very high number of low-abundance bacterial taxa in zoo apes. It is plausible to assume that those organisms are introduced by the food provided in the zoo. As such they might represent only transient species, given that the indigenous microflora is usually able to defend its ecological niches successful against foreign bacteria [33]. This barrier against foreign bacteria is based on interactions between the indigenous microflora and the immune system, which in turn is the result of long-term coevolution in animals [34]. However, the interplay between the immune system and indigenous microflora might work best in the natural habitat, where it evolved. The conspicuous high number of low-abundance bacteria in zoo apes might indicate that this balance is (at least partially) disrupted and that eventually at least some of the novel bacteria may be able to occupy distinct oral niches. As such, our results call into question conclusions about the microbiome of species that are based on analyses of zoo animals [5,35]. To be sure, studies based on zoo animals have largely focused on the gut microbiome, as revealed by analyses of fecal material, which may be more buffered from outside environmental influences than the saliva microbiome. Nonetheless, the oral cavity is an important entry point for bacteria into the gut, and hence it is quite probable that the gut microbiome would be similarly influenced by the zoo environment. Inferences based on the analysis of microbiomes of zoo or other captive animals therefore should, whenever possible, be buttressed by analysis of samples from individuals in the wild [9,10]. In sum, the comparative analyses of the saliva microbiome from our nearest living relatives, chimpanzees and bonobos, greatly enrich our knowledge of and provide new perspectives on the saliva microbiome of our own species.

## Methods

### Samples

Saliva samples were collected from bonobos (*Pan paniscus*) and staff members at the Lola ya Bonobo Sanctuary, Kinshasa, Democratic Republic of Congo (DRC), and from chimpanzees (*Pan troglodytes*) and staff members at the Tacugama Chimpanzee Sanctuary, Freetown, Sierra Leone (SL). The chimpanzee and bonobo samples were collected while the animals were anesthetized (via injection) for annual medical examinations; swabs were used to absorb saliva. Bonobo samples were imported under CITES permit E-02526/09, while chimpanzee samples were imported under CITES permit E-01349/09. Samples from apes at the Leipzig Zoo were collected noninvasively, by using swabs to absorb saliva from the mouth. Swabs from both sanctuary and zoo apes were immediately added to lysis buffer [36] and kept at ambient temperature for up to one month before extraction. Human volunteers spit up to 2 mL of saliva into tubes containing 2 mL lysis buffer [36]. While the oral health of donors at the time of sampling was not investigated in detail, no ape or human donor was suffering from obvious oral lesions or severe dental decay, and to the best of our knowledge no ape or human was being treated with antibiotics at the time of sampling. Estimated ages of the apes ranged from 5–20 years, and of the human donors from 20–40 years. Informed consent was obtained from all human donors. As relevant ethical review boards did not exist in the DRC and Sierra Leone at the time of sampling, the collection of human samples was approved by the directors of the sanctuaries, and by the Ethics Commission of the University of Leipzig Medical Faculty.

### DNA extraction and PCR

DNA was extracted as described previously [36]. Two variable segments of the microbial 16S rRNA gene, V1 and V2, were amplified in a single ~350 bp product (corresponding to positions 8–361 of the *E. coli* K12 reference sequence), using the forward primer for V1 and the reverse primer for V2 and the PCR conditions published elsewhere [37].

### Sequencing on the genome Sequencer FLX platform

The PCR products were processed for parallel-tagged sequencing on the Genome Sequencer FLX platform, as described elsewhere [38]. Briefly, sample-specific barcode sequences were ligated to the PCR products, and DNA concentrations were assessed with a Mx3005P™ qPCR System (Stratagene). Samples were then pooled in equimolar ratios to a total DNA amount of 440 ng. The pooled DNA was subsequently amplified in PCR-mixture-in-oil emulsions and sequenced on a Genome Sequencer FLX /454 Life Sciences sequencer (Branford CT), according to the manufacturer’s protocol.

### Data analysis

The initial sequence reads were filtered to remove low-quality sequences and artifactual sequence reads (i.e., reads containing two or more different tags, no tags, primers in the middle of sequence reads, or lacking a primer sequence). After removing sequences less than 200 bp in length (as these may not give reliable results), there were 48,168 sequence reads used in the analysis. These sequence reads have been deposited in GenbankSequence Read Archive (SRA) SRP015938. A genus was assigned to each sequence by comparing the filtered sequences against the Ribosomal Database Project [16] using the online program SEQMATCH (http://rdp.cme.msu.edu/seqmatch/seqmatch_intro.jsp) and a threshold setting of 90%. Diversity statistics and the apportionment of variation based on the frequency distribution of genera within and between individuals were calculated with the Arlequin 3.1 software [39]. Spearman’s rank correlation coefficients, sharing (Venn) diagrams, and Analysis of Similarity (ANOSIM) [40] were calculated with the R package. Rarefaction analysis was carried out using the Resampling Rarefaction 1.3 software (http://strata.uga.edu/software/). Partial correlation analysis was carried out with the GeneNet package [41]. For the UniFrac analysis, the sequences were aligned with the Infernal 1.0 program [42] and a phylogenetic tree was constructed under a generalized time reversible (GTR) model with the FastTree software [43]. Fast UniFrac [19] was then used to compare the microbial communities, compute the distance matrix, and generate the cluster tree. The phylogenetic tree from FastTree was also used to calculate Faith’s Phylogenetic Diversity [20] using the “picante” package in R [44]. The OTU networks were constructed from the sequences aligned with Infernal 1.0 by using tools provided by the RDP website to first cluster all sequences that were 97% or more similar (based on a minimum overlap of 25 bases) into OTUs (to account for sequencing errors). We then used the Cytoscape 2.8 software [45] to generate and visualize the networks. Briefly, each individual is considered a Source node and each OTU is a Target node. Target nodes were linked to Source nodes in a bipartite network, with connections between Sources and Targets modeled as springs; both Source and Target nodes are placed in such a way as to minimize the forces across the network.

## Competing interests

The authors declare that they have no competing interests.

## Authors’ contributions

MS designed the study. CA, RMG, MH, and AF collected the samples. DQ carried out the laboratory work. JL, IN, ML, and HPH analyzed the data. MS, JL, and HPH wrote the manuscript. All authors read and approved the final manuscript (with the exception of IN, who read and approved a preliminary version).

## Supplementary Material

Additional file 1: Table S1 Number of reads assigned to each genus in sanctuary apes and human workers. Click here for file

Additional file 2: Figure S1 Rarefaction analysis. **Figure S2.** Heat plot of the frequency of each microbial genus in the saliva microbiome of each individual. **Figure S3.** Partial correlation analysis of associations among bacterial genera from humans and from apes. **Figure S4.** Heat plot of correlation coefficients, based on the frequency of bacterial genera in the saliva samples from sanctuary apes and human workers. **Figure S5.** Average UniFrac distances between different groups. **Figure S6.** Faith’s PD, which is a measure of the within-group diversity based on bacterial OTUs. Click here for file

Additional file 3: Table S2 Bacterial phyla detected in fecal samples from humans, chimpanzees and bonobos from a previous study [9] and in saliva samples from the present study. Click here for file

Additional file 4: Table S2 Number of reads assigned to each genus for zoo apes. Click here for file

Additional file 5: Table S4 Number (above diagonal) and percentage (below diagonal) of OTUs shared between different groups of apes and humans. Click here for file

Additional file 6: Table S5 Bacterial genus assigned to each OTU, and number of sequences from each group assigned to each OTU. Click here for file
