# Supplementary material for: The saliva microbiome of Pan and Homo
Source: BMC Microbiol. 2013 Sep 11;13:204. doi: 10.1186/1471-2180-13-204 (PMC3848470; doi:10.1186/1471-2180-13-204)
Supplement: Additional file 2: Figure S1 — Rarefaction analysis. Figure S2. Heat plot of the frequency of each microbial genus in the saliva microbiome of each individual. Figure S3. Partial correlation analysis of associations among bacterial genera from humans and from apes. Figure S4. Heat plot of correlation coefficients, based on the frequency of bacterial genera in the saliva samples from sanctuary apes and human workers. Figure S5. Average UniFrac distances between different groups. Figure S6. Faith’s PD, which is a measure of the within-group diversity based on bacterial OTUs. [file 1471-2180-13-204-S2.doc]

**Figure S1**. Rarefaction analysis. Top, based on bacterial genera; bottom, based on OTUs. The X-axis is the number of reads and the Y-axis is the number of different genera/OTUs detected; the error bars indicated the 95% confidence intervals.

**Figure S2.** Heat plot of the frequency of each microbial genus in the saliva microbiome of each individual. Each row is an individual, and each column is a genus, numbered according to Supplementary Tables S1 and S3. Top: Bonobos, Chimpanzees, DRC Humans, and SL Humans; Bottom: zoo apes.


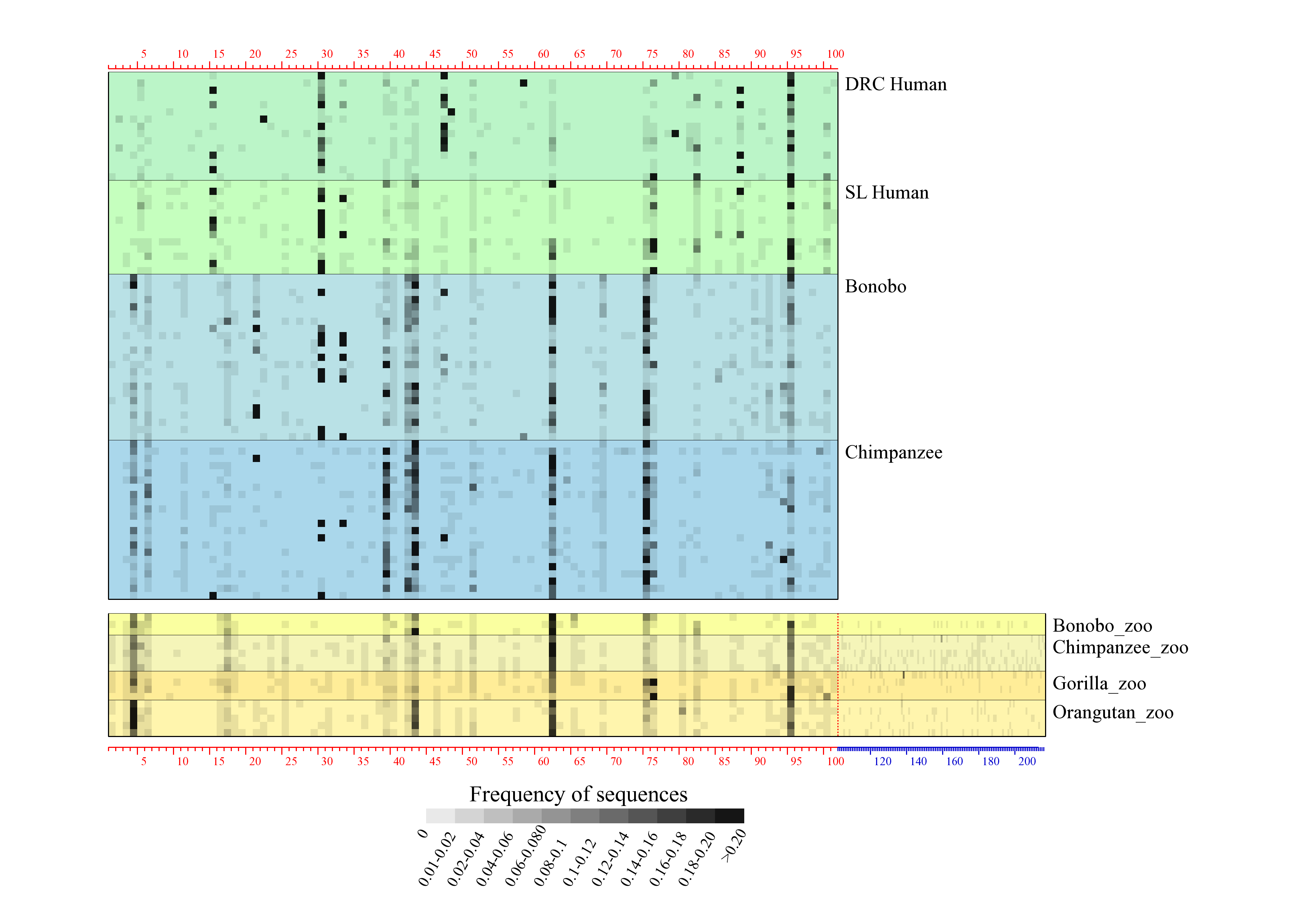


**Figure S3.** Partial correlation analysis of associations among bacterial genera from humans and from apes. Partial correlation coefficients were calculated from the frequency abundance of genera in each individual and their significance determined by permutations. The network shows the significant correlations among the 28 most abundant genera (with a frequency of at least 0.5%); the solid lines indicate positive correlations and the dashed lines represent negative correlations, with the darkness of the lines indicating the strength of the correlation. **A**, network for genera in humans; **B**, network for genera in apes.

**A**


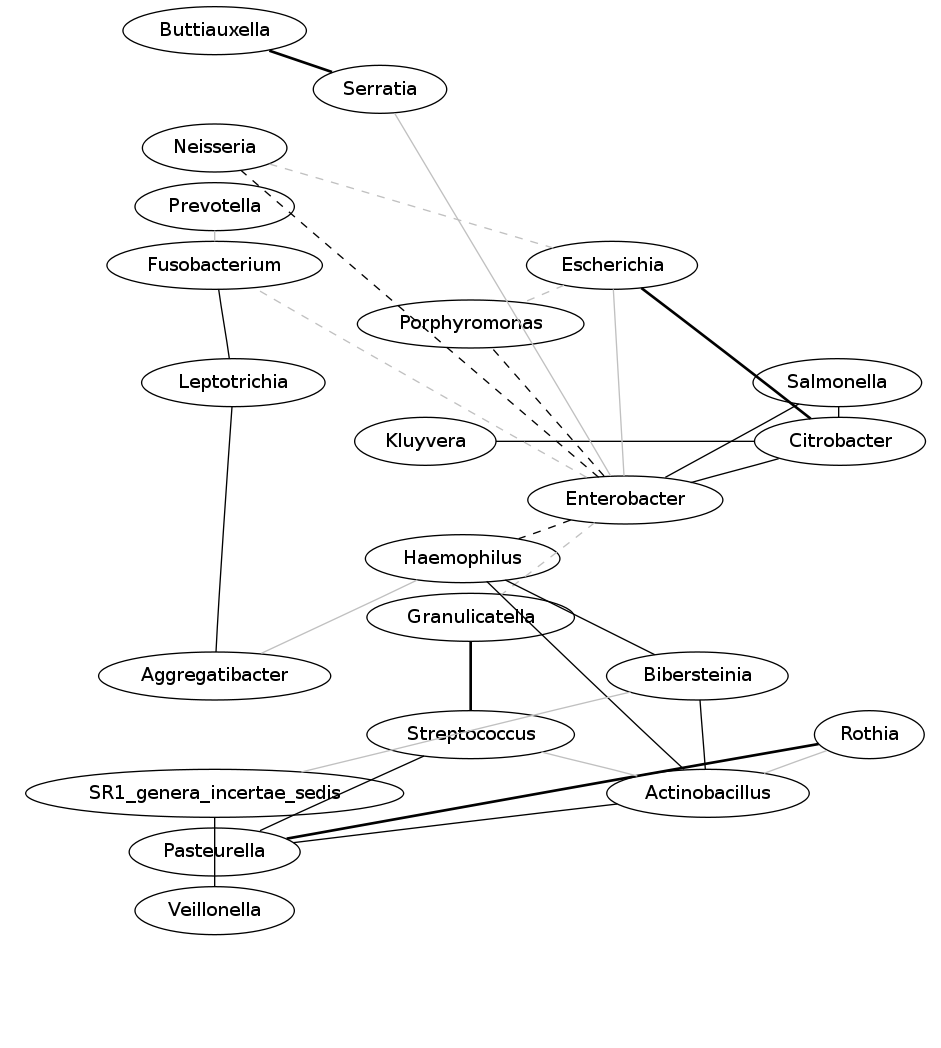


**B**

**
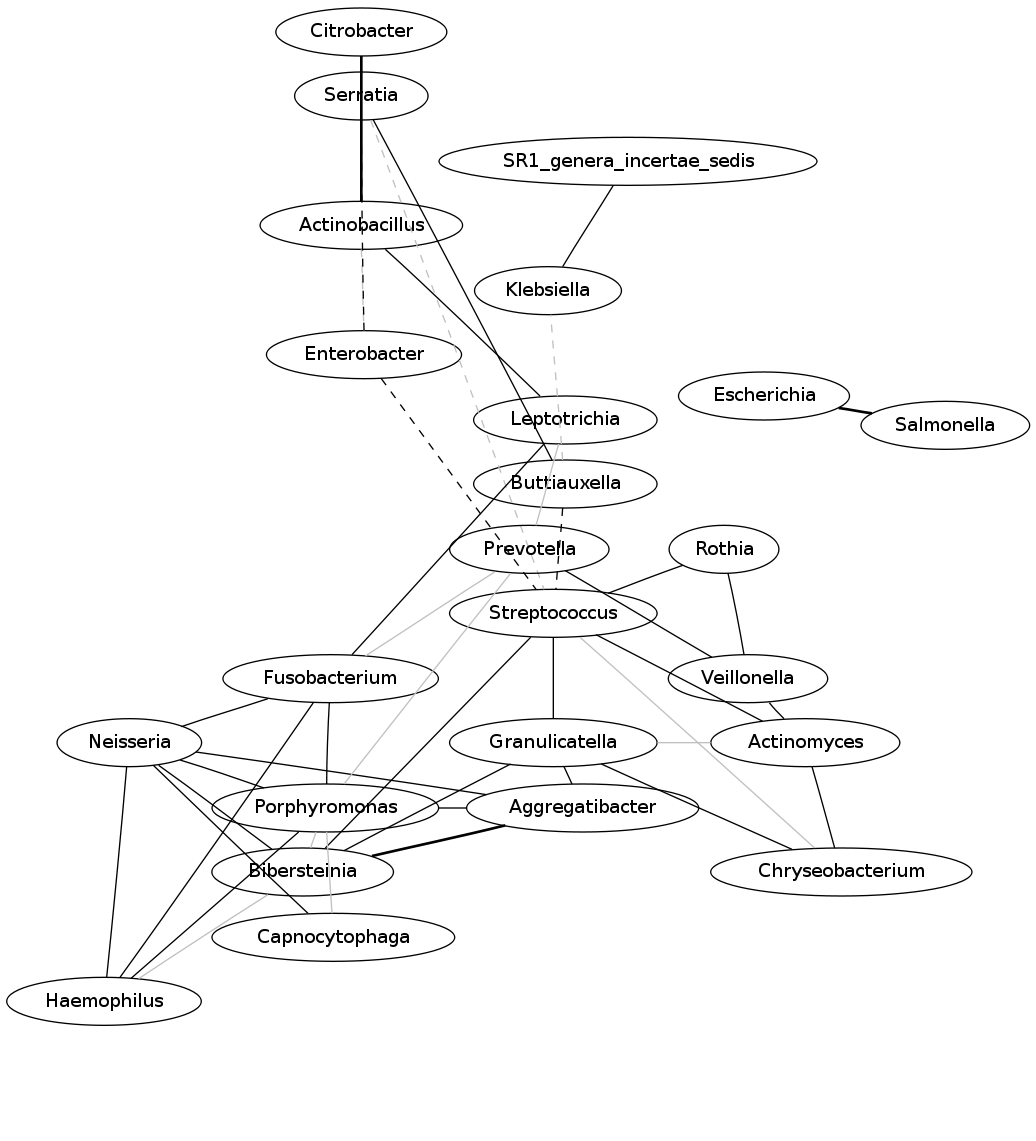
**

**Figure S4.** Heat plot of correlation coefficients, based on the frequency of bacterial genera in the saliva samples from sanctuary apes and human workers.


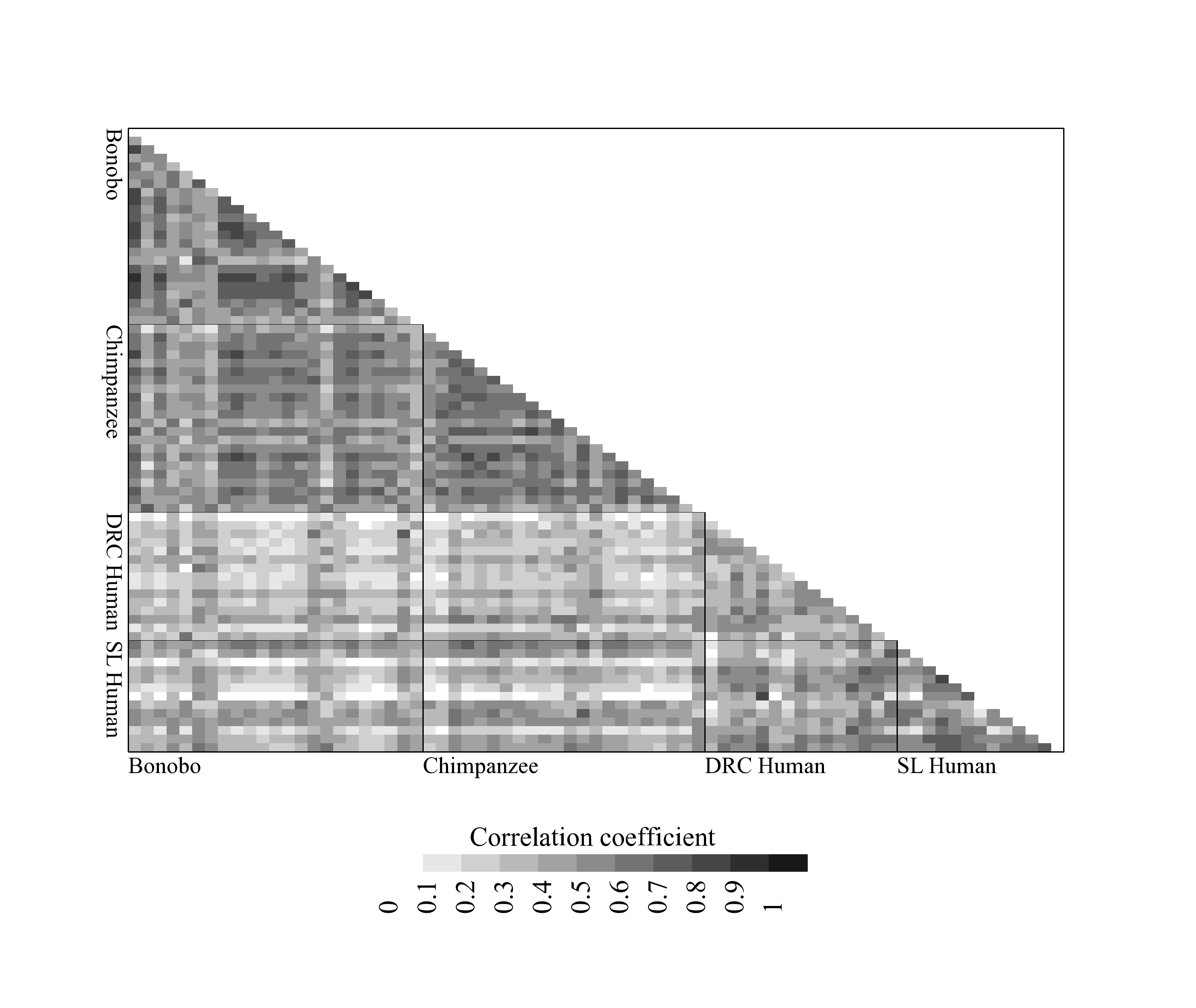


**Figure S5**. Average UniFrac distances between different groups. The top bars show the average distances between different groups of species, while the bottom bars show the average distance between the four zoo ape species (“Zoo apes”), the two human groups (“Humans”), and the two wild ape species (“Wild apes”). Significance between comparisons is indicated as *p<0.05, **p<0.01, as assessed by a Mann-Whitney U test.

**Figure S6**. Faith’s PD, which is a measure of the within-group diversity based on bacterial OTUs. Error bars indicated one standard error.
